# Retinoic Acid Signaling in B Cells Is Required for the Generation of an Effective T-Independent Immune Response

**DOI:** 10.3389/fimmu.2016.00643

**Published:** 2016-12-23

**Authors:** Ellen Marks, Carla Ortiz, Eirini Pantazi, Charlotte S. Bailey, Graham M. Lord, Thomas J. Waldschmidt, Randolph J. Noelle, Raul Elgueta

**Affiliations:** ^1^Department of Mucosal Immunology, Division of Transplantation Immunology & Mucosal Biology, Guy’s Hospital, King’s College London, London, UK; ^2^Department of Immune Regulation and Intervention, Division of Transplantation Immunology & Mucosal Biology, Guy’s Hospital, King’s College London, London, UK; ^3^Interdisciplinary Graduate Program in Immunology, Carver College of Medicine, The University of Iowa, Iowa City, IA, USA; ^4^Department of Microbiology and Immunology of Dartmouth Medical School, Norris Cotton Cancer Center, Lebanon, NH, USA

**Keywords:** retinoic acid, B cell, peritoneum, marginal zone, immunoglobulins

## Abstract

Retinoic acid (RA) plays an important role in the balance of inflammation and tolerance in T cells. Furthermore, it has been demonstrated that RA facilitates IgA isotype switching in B cells *in vivo*. However, it is unclear whether RA has a direct effect on T-independent B cell responses *in vivo*. To address this question, we generated a mouse model where RA signaling is specifically silenced in the B cell lineage. This was achieved through the overexpression of a dominant negative receptor α for RA (dnRARα) in the B cell lineage. In this model, we found a dramatic reduction in marginal zone (MZ) B cells and accumulation of transitional 2 B cells in the spleen. We also observed a reduction in B1 B cells in the peritoneum with a defect in the T-independent B cell response against 2,4,6-trinitrophenyl. This was not a result of inhibited development of B cells in the bone marrow, but likely the result of both defective expression of S1P_1_ in MZ B cells and a defect in the development of MZ and B1 B cells. This suggests that RARα expression in B cells is important for B cell frequency in the MZ and peritoneum, which is crucial for the generation of T-independent humoral responses.

## Introduction

B cells play an essential role in the protection against infection, contributing to both innate and adaptive immune responses. They are derived from the bone marrow and undergo a developmental process that results in terminally differentiated plasma cells, acquiring antibody producing capacity, which mediates the neutralization and removal of microbes and infected cells (1). When exposed to a cognate antigen, B cells are capable of responding in a T cell-independent manner *via* their differentiation into short-lived plasma cells. These short-lived plasma cells produce a limited array of Ig isotypes; in the first instance, they produce IgM antibodies and then, to a lesser extent, produce IgG antibodies. In addition, the antibodies produced by short-lived plasma cells have not undergone affinity maturation, resulting in low specificity of responses (2). The initial T-independent response normally dissipates after a week (2).

An optimal T-independent B cell response requires two different subsets of B cells; marginal zone (MZ) B cells and B1 B cells. MZ B cells are produced in the spleen and result from the differentiation of immature bone marrow-derived B cells (3). MZ B cells are derived from immature Transitional 2 (T2) B cells (4) in a process mediated by high expression of delta-like 1 expressed in splenic venules (5) and NF-κB signaling (6).

In contrast, it is known that B1 B cells are produced in the peritoneal and pleural cavities (3); however, the signaling mechanisms involved in the development of this subset are not fully understood. Nonetheless, it is clear that B1 B cells are developed by the first weeks following birth (7) and maintained during adulthood by self-renewal (8).

B1 B cells play an important role in IgA gut humoral responses following migration of these cells from the peritoneum to the lamina propria of the intestine. In the lamina propria, B1 B cells differentiate into polyspecific IgA-plasma cells in a process that is dependent on IL-5 (9). Thus, MZ and B1 B cells are key to the production of natural antibodies and maintenance of tissue homeostasis.

Several factors regulate B-cell growth, survival, maturation, and migration. It has been shown that retinoic acid (RA), a product derived from vitamin A, plays an important role in these events. Vitamin A deficiency drastically increases the mortality rate as a result of measles infection (10) or diarrhea (11). In addition, supplementation with vitamin A reduces the morbidity of these and others infectious diseases (12), suggesting that vitamin A plays an important role in T and B cell-mediated immunity. In animal models, it has been demonstrated that vitamin A deficiency reduces antibody titers against tetanus toxin, which is a T-dependent B-cell response (13, 14). Vitamin A deficiency has also been shown to decrease antigen-specific IgG responses (15, 16). Similarly, lack of vitamin A reduces the levels of antibodies in T-cell-independent type 2 (TI2) responses when pneumococcal polysaccharide is used as an antigen (17). Antibody titers are rescued after the administration of vitamin A, indicating that there is a correlation between levels of vitamin A and the production of an effective TI2 response (18).

Because of the significant effects of vitamin A on B cell differentiation, it has been evaluated as an adjuvant for augmentation of the immune response. In fact, RA in combination with IL-15 can induce potent cellular and humoral responses (19). In addition, it has been shown that the T cell-independent type 1 (TI1) response is normal in vitamin A-deficient rats, whereas TI2 is abrogated (20). This suggests that the reduction in antibody production is due to a defect in the response to specific antigens rather than an intrinsic defect in the synthesis of antibodies. Moreover, the lack of an effective TI2 response could be explained by the reduction in MZ B cells and B1 B cells in vitamin A-deficient mice (21). Thus, RA may play an important role in TI2 responses.

The development of B cells is also regulated by RA (22). B cell progenitors treated with RA differentiate into mature B cells, reducing the time of differentiation without affecting the proliferation of the progenitors (22). These results were corroborated *in vivo* using mice treated with all-trans RA (ATRA). Mice treated with ATRA display increased numbers of mature B-cells in the bone marrow and spleen, despite exhibiting a decreased number of B-cells precursors. RA acts through the RA receptor α (RARα) to induce Pax5, a key transcription factor in the maturation of B cells and a repressor of plasma cell differentiation (22, 23).

Here, we highlight the importance of RA signaling in the development of T cell-independent B cell immune responses. Using a genetic approach, by overexpressing a dominant-negative form of RARα specifically in the B cell compartment (24), we demonstrate that RA signaling in B cells is required for the distribution of MZ and B1 B cells and to mount an effective T-independent immune response.

## Materials and Methods

### Ethics Statement

These studies were approved and conducted in accredited facilities in accordance with The Home Office UK Animals (Scientific Procedures) Act 1986 (Home Office license number PPL 70/7102).

### Mice and Immunizations

C57BL/6 (H-2b) mice were purchased from Harlan Laboratory. CD19^Cre^ mice were purchased from the Jackson Laboratory. The dominant negative (dn) RARα mice have been previously described (25). We generated the dnRAR^fl/fl^CD19^Cre/+^ (heterozygous form for CD19^Cre^), in a manner similar to previous reports (24). The CD19^Cre/+^ control mice are haplosufficient and had a normal B cell compartment as previous described (24). To evaluate T-independent immune responses, mice aged 8–10 weeks were immunized with 50 µg of the hapten (Tri-4-hydroxy-3-nitrophenyl) acetyl (TNP)-Ficoll (Biosearch Technology). To study T-dependent humoral immune responses, 50 µg of TNP-KLH (Biosearch Technology) adsorbed in alum was injected i.p. in a volume of 200 µl. These studies were approved and conducted in accredited facilities in accordance with the United Kingdom Animals (Scientific Procedures) Act 1986 (Home Office license no. PPL 70/7102). All animals were cohoused and maintained in a specific pathogen-free facility at King’s College London.

### Cell Preparation

To analyze B cells, single-cell suspensions of lymphocytes were prepared from spleens by mechanical disruption in DPBS followed by passing the cells through a 70 µm cell strainer. Cells were collected by centrifugation (5 min, 500 × *g*, 4°C) and red blood cells were lysed (2 min, 37°C) using RBC Lysis buffer (BioLegend). Total number of cells and cell viability were determined using a hemocytometer and trypan blue. For isolation of peritoneal cells, the peritoneal cavity was flushed with 5 ml warm (37°C) PBS, 2% BSA, 2 mM EDTA, 0.02% sodium azide, and 10 U/ml Heparin.

### Antibodies and Flow Cytometry

Antibodies against the following antigens were used: anti-mouse B220 (clone 6B2), CD38 (clone 90), IgM (clone 11–41), CD24 (clone M1/69), CD21/35 (clone 7E9), CD23 (clone B3B4), and IgD (clone 11-26c) from BD Biosciences. Anti-mouse CD5 (clone 53-7.3) and CD11b (clone M1/70) from Biolegend. Anti-mouse CD11a (integrin αL, clone M17/4), CD18 (integrin β2, clone M18/2), CD29 (integrin β1, clone HMb1-1), and CD49d (integrin α4, clone R1-2) from eBioscience. Murine cells were resuspended in DPBS + 3% FCS and stained for 30 min at 4°C with different anti-mouse antibodies in the presence of anti-mouse TruStain fcX (Biolegend). Control samples were labeled with matched isotype control antibodies. The samples were resuspended in Sytox Blue Dead Cell Stain (1:1,000 in DPBS; Invitrogen) before acquisition. Samples were acquired by flow cytometry (BD LSRFortessa cell analyser, Becton Dickinson) with FACSDiva software v6.2 (Becton and Dickinson) and data were analyzed using FlowJo (Tree Star) software.

### Purification of B Cell Subsets

B cells were purified from the spleens of dnRARαCD19^Cre^ or littermate control mice by negative selection using CD43 microbeads (Miltenyi Biotech) according to the manufacturer’s instructions. Purified splenic B cells were stained with anti-CD21/35, anti-CD24, anti-CD23, and anti-IgM as described previously (26). Sytox was added to exclude dead cells. Cells were sorted by BD FACSAria II (BD Biosciences). In some cases, MZ B cells were cultured with 1µM of LE135 (Tocris Biosciences), an antagonist for RAR receptors, for 24 h as previously described (27).

### Real-time PCR

Total RNA was isolated from purified B cells using MiniKit RNeasy columns (QIAGEN) with a DNase-I treatment step. One microgram of DNA-free RNA was reverse transcribed to cDNA using Omniscript RT (QIAGEN). TaqMan gene expression assays containing FAM dye-labeled TaqMan MGB probe was used for mouse S1pr1 (Mm02619656_s1), RARα (Mm01296312_m1), RARβ (Mm01319677_m1), and RARγ (Mm00441091_m1) in multiplex with primer-limited assays for glyceraldehyde-3-phosphate dehydrogenase (GAPDH) endogenous control containing VIC/MGB probes. Real-time quantification was performed using TaqMan gene expression master mix on a Bio-Rad CFX96 optical reaction module on a C1000 thermal cycler. Data were analyzed using CFX Manager Software (Bio-Rad).

### Microscopy/Immunofluorescence

Tissue was then snap frozen in OCT Tissue-Tek freezing medium (Sakura). Cryostat sections of 7 µm were air dried, fixed with acetone, and washed in DPBS. Blocking was performed with 20% normal horse serum (PAA Laboratories Inc.) for 15 min at room temperature. Sections were incubated with biotin-conjugated anti-mouse IgM (clone 11–41, BD Biosciences), followed by streptavidin Alexa Fluor 594 (Life Technologies) or FITC-conjugated anti-mouse monocyte and macrophage (MOMA; Abcam). Negative controls were no primary antibody or stained with isotype-matched antibody. Images were acquired on an Olympus BX51 microscope using Micro-Manager software (Vale Laboratory).

### Chemotaxis Assays

Migration assays were performed as previously described (28). FACS-sorted MZ B cells were treated with 1 µM of LE135, or left untreated, before analysis using a chemotaxis assay. Transwell assays were performed in duplicate, using 100 nM for S1P and 250 nM for SDF-1 as a chemoattractant. Two mice were pooled in each assay, in three independent experiments. It was not possible to perform *ex vivo* chemotaxis assays with cells from dnRARαCD19^Cre^ (unpublished data).

### ELISA Analysis

For ELISA analysis, plates were coated with 10 mg/ml TNP-BSA overnight in PBS, blocked with PBS + 5% FBS, washed, and then 1:2,000 diluted serum added at serial 1:2 dilutions. Anti-TNP IgG3 (provided by Thomas J. Waldschmidt) and anti-TNP IgM (BD Bioscience) was included on each plate as a reference between plates and between experiments and used to generate a standard curve. Antibody levels were detected with AP-conjugated IgG3 and IgM (Southern Biotechnology Associates) and developed with 1 mg/ml para-nitrophenylphosphate (Sigma-Aldrich) in 0.05 mM sodium carbonate buffer.

### ELISPOT Analysis

Single-cell preparations were obtained from the spleen and the number of TNP-IgM, TNP-IgG_3_, and TNP-IgG_1_ antibody-secreting cells (ASCs) were determined by ELISPOT as previously reported (29, 30).

### Statistics

Results are expressed as mean ± SD. Comparisons between groups were performed using *T*-test for two groups, or one-way ANOVA and a Dunnett or Bonferroni posttest for more than two groups. Analyses were performed using GraphPad Prism software.

## Results

### RA Signaling in B Cells Affects the Development of MZ B Cells

**Figure 1:**
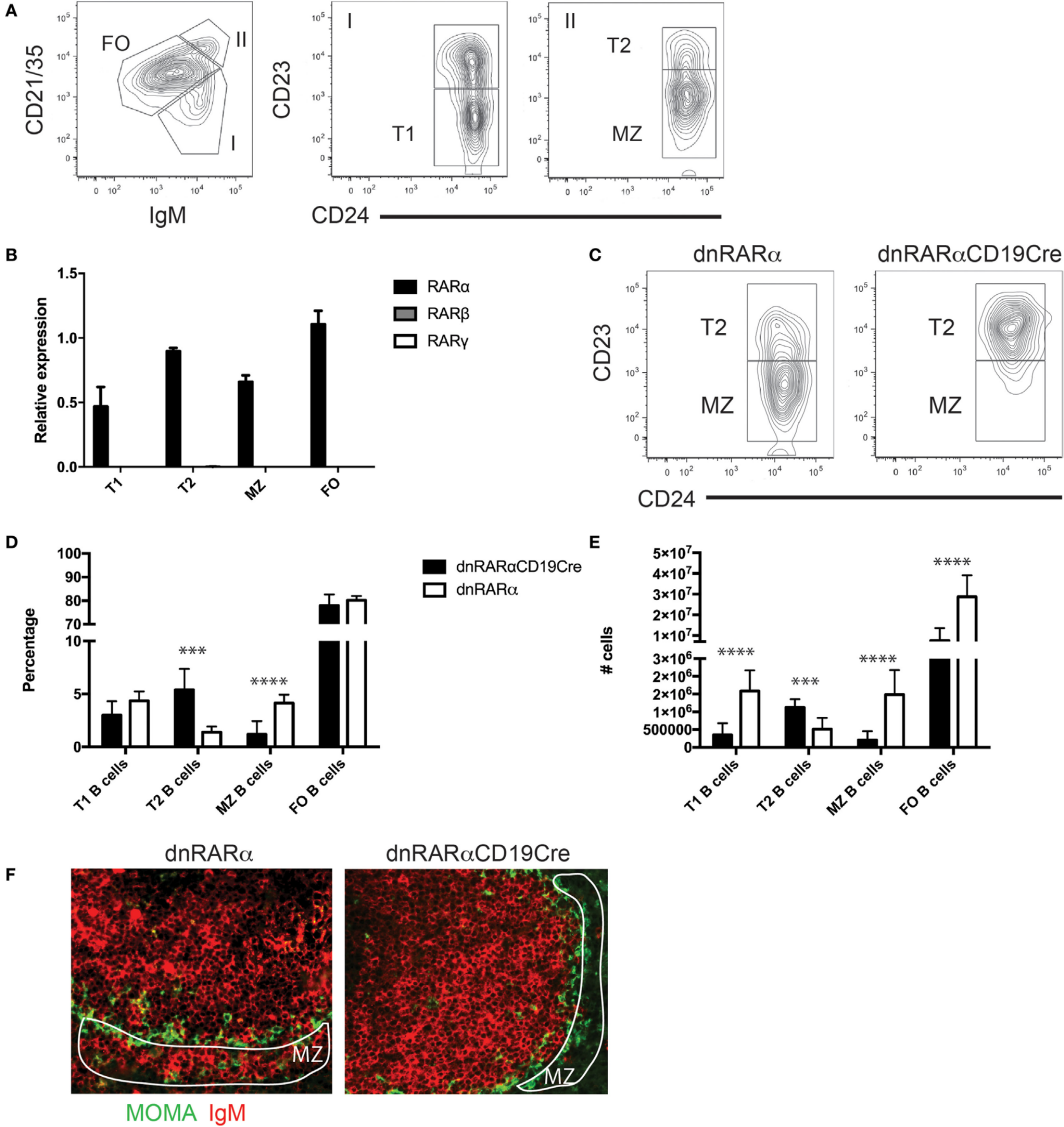
**The inhibition of RA signaling in B cells impairs the development of MZ B cells**. **(A)** Gating strategy for the different subsets of splenic B cells. FO B cells B220^+^ (CD21/35^int^ IgM^int^ CD24^+^ CD23^+^), T1 B cells (B220^+^ CD21/35^low^ IgM^+^ CD24^+^ CD23^−^), T2 (B220^+^ CD21/35^+^ IgM^+^ CD24^+^ CD23^+^), and MZ B cells (B220^+^ CD21/35^+^ IgM^+^ CD24^+^ CD23^−^). Cells were pre-gated on live, B220^+^ cells. **(B)** Quantification of RAR transcript levels in the indicated splenic B cell subsets. **(C)** A representative flow cytometry contour plot of MZ B cells and Carsetti T2 B (B220^+^ CD21/35^+^ IgM^+^ CD24^+^ CD23^+^) cells from spleen of dnRARαCD19^Cre^ (right panel) or littermate controls (left panel). Cells were pre-gated on live, B220^+^ CD21/35hi IgM^+^ cells. **(D)** Percentage and **(E)** absolute number of follicular (FO), marginal zone (MZ), transitional 1 (T1), and transitional 2 (T2) B cells from spleen of dnRARαCD19^Cre^ or littermate controls. Three independent experiments with at least seven mice/group. **(F)** Histology of the spleen from dnRARαCD19^Cre^ (right panel) or littermate controls (left panel) stained with MOMA-FITC (Green) and IgM-biotin plus streptoavidin-alexafluor455 (Red) for MZ analysis. Encircled white area indicates the MZ. The original magnification is 20×. Representative of three independent experiments with three mice/group. ***p < 0.001 and ****p < 0.0001.

To evaluate the role of RA signaling in T-independent humoral responses, we first evaluated the expression of the different RAR isoforms in splenic B cell subpopulations. Figure 1A shows the gating strategy used to electronically sort splenic B cell subsets, T1, T2, MZ, and FO splenic B cells, for analysis of RNA expression levels of RAR isoforms (all >95% purity). Our results showed that these subsets of splenic B cells exclusively express RARα, but not the β and γ isoforms (Figure 1B).

To study the functional role of RARα in splenic B cell function, we used mice engineered to express a dominant negative form of the RARα (RARα403) targeted to the ROSA26 locus downstream of a *lox*P-flanked “*stop*” cassette. Upon breeding with mice expressing Cre recombinase (in this case CD19^Cre^), RA signaling is abrogated in the CD19^+^ B cell compartment (hereafter denoted as dnRARαCD19^Cre^). We have previously used this approach and demonstrated that dnRARα is expressed exclusively in B cells from dnRARαCD19^Cre^ mice and is fully functional (24).

We then characterized whether RA signaling in B cells has an impact on the development of splenic B cell subsets *in vivo*. We observed an increase in the percentage (Figures 1C,D) and absolute number of T2 B cells in the dnRARαCD19^Cre^ mice compared with littermate controls (Figure 1E). In contrast, we observed a reduction in the percentage and absolute number of MZ B cells in the spleen of dnRARαCD19^Cre^ mice compared to control mice (Figures 1C–E). We then analyzed the splenic architecture by histology in dnRARαCD19^Cre^ mice. The data shown in Figure 1F demonstrates a reduction in MZ B cells in the spleen of dnRARαCD19^Cre^ mice compared to littermate controls. In addition, no difference was found in MHC-II, CD80 or CD86 expression in B cells from dnRARαCD19^Cre^ and littermate controls (data not shown). Together, these data suggest that RA signaling in B cells is necessary for the development of MZ B cells.

### RA Signaling Regulates S1P_1_ Expression in MZ B Cells

**Figure 2:**
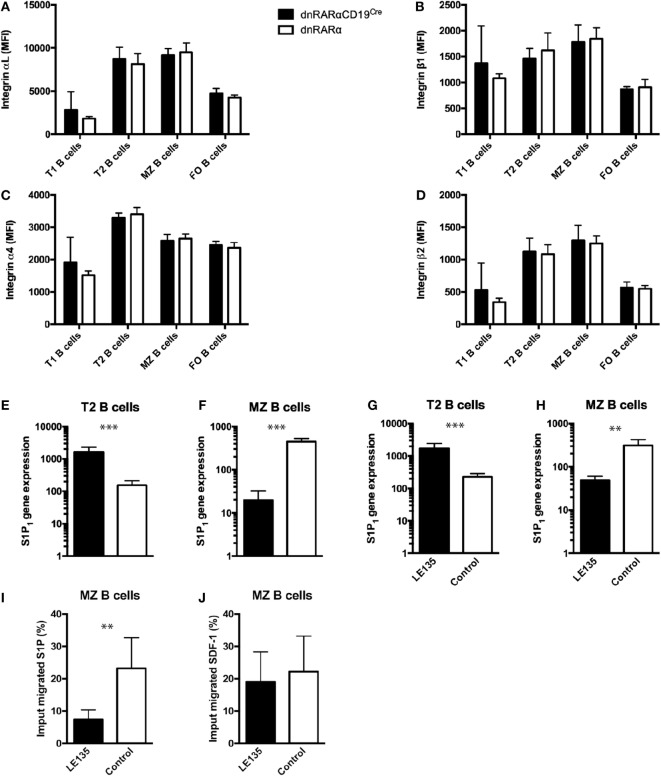
**RA signaling in B cells regulates the expression of S1P_1_**. **(A–D)** Mean fluorescence intensity of integrins **(A)** αL, **(B)** β1, **(C)** α4, and **(D)** β2 in different splenic subsets B cells from dnRARαCD19^Cre^ or littermate controls. **(E,F)** Quantification of S1P_1_ transcript levels in the **(E)** T2 B cells and **(F)** MZ B cells from dnRARαCD19^Cre^ or control mice. Three independent experiments with at least seven mice/group. **(G,H)** Quantification of S1P_1_ transcript levels in wild type **(G)** T2 B cells and **(H)** MZ B cells treated with 1 µM of LE135m, or untreated. **(I,J)** Chemotaxis assay of wild type MZ B cells treated with 1 µM of LE135, or untreated, to **(I)** 100 nM of S1P or to **(J)** 250 nM SDF-1. Three independent experiments with at least two to three mice/group. ***p < 0.001 and **p < 0.01.

It has been previously reported that the integrins α_L_β_2_ and α_4_β_1_ facilitate long-term retention of B cells in the MZ (31). We assessed whether RA signaling in B cells affects the expression of these integrins in MZ B cells or T2 B cells. Our results showed no difference in the expression of these integrins in any of the studied B cell subsets (Figures 2A–D). Another molecule involved in the migration of B cells to the MZ is S1P_1_ (28). We evaluated S1P_1_ expression on T2 and MZ B cells from dnRARαCD19^Cre^ mice and observed that it was drastically increased in T2 B cells (Figure 2E), but reduced in MZ B cells from dnRARαCD19^Cre^ mice compared with littermate controls (Figure 2F). Together, these results suggest that RA signaling is necessary to regulate the expression of S1P_1_ in T2 and MZ B cells.

In order to evaluate whether RA signaling in MZ B cells affects migration to S1P, we sought perform chemotaxis assays using MZ B cells from dnRARαCD19^Cre^. However, due to low cell numbers, it was not possible to perform *ex vivo* chemotaxis assays with cells from dnRARαCD19^Cre^ (unpublished data). Instead, we compared wild-type MZ B cells treated with LE135, an antagonist for RAR signaling, to untreated wild-type MZ B cells (27). First, we evaluated whether LE135 affects S1P_1_ expression in B cells. As observed in T2 and MZ B cells from dnRARαCD19^Cre^ mice (Figures 2E,F), LE135 increased the expression of S1P_1_ in T2 B cells (Figure 2G) whereas S1P_1_ expression in MZ B cells treated with LE135 was reduced (Figure 2H). In addition, LE135 treatment drastically reduced the ability of MZ B cell to respond to S1P (Figure 2I), without affecting the chemotaxis to SDF-1 (Figure 2J). Together, these results suggest that RA signaling in B cells plays an important role in the expression of S1P_1_ and induces S1P-mediated chemotaxis in MZ B cells.

### RA Signaling in B Cells Is Necessary for B1 B Cell Generation and for T Cell-Independent Immune Responses

**Figure 3:**
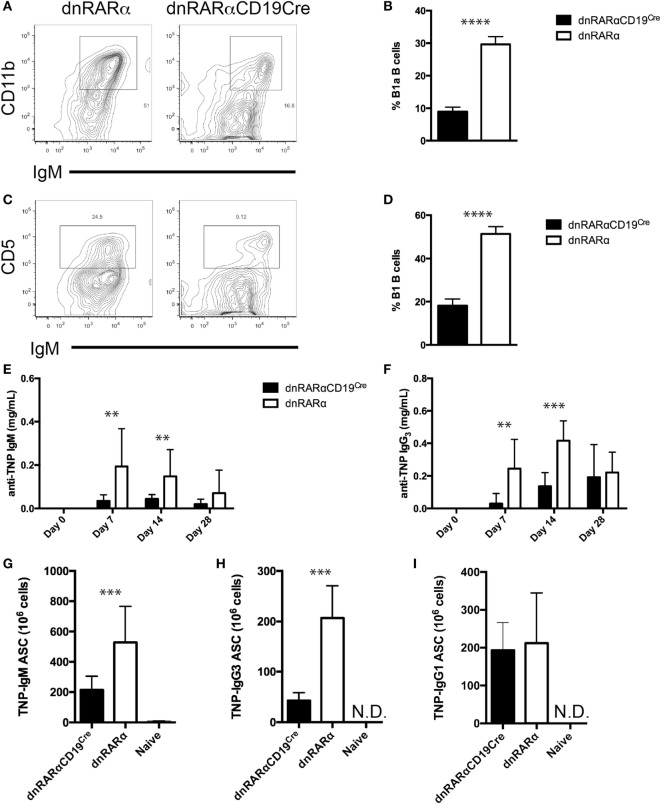
**B1 B cells are reduced and the T-independent humoral response is inhibited in the absence of RA signaling in the B cell compartment**. **(A,C)** Representative contour plot of **(A)** B1 (CD11b+ IgM+) and **(C)** B1a (CD5+ IgM+) B cells from dnRARαCD19^Cre^ and control mice are shown. The numbers in the corners of the plots represent the percentage of total B cells. The cells were pre-gated on live B220^+^ cells. Quantification of **(B)** B1 B cells and **(D)** B1a B cells in peritoneal lavage from dnRARαCD19^Cre^ and control mice. **(E,F)** Quantification of **(E)** TNP-IgM and **(F)** TNP-IgG_3_ titers in serum by ELISA. **(G–I)** Number of **(G)** TNP-IgM, **(H)** TNP-IgG_3_, and **(I)** TNP-IgG_1_ antibody-secreting cell, as detected by ELISPOT, in the spleen are shown. Three independent experiments with at least eight mice/group. **p < 0.01, ***p < 0.001, and ****p < 0.0001.

RA signaling in B cells is important for IgA plasma cell differentiation and to maintain IgA titers in the gut (24). In addition, it has been proposed that peritoneal B cells migrate from the peritoneum to the lamina propria of the small intestine to contribute to IgA antibody levels in the gut (32, 33). Thus, we studied whether RA signaling in B cells could also affect the generation of peritoneal B cells. We observed a 60% reduction in the proportion of B1a and B1b B cells in the peritoneum of dnRARαCD19^Cre^ mice compared to controls (Figures 3A–D), which suggests that RA signaling is necessary in B cells for the generation or migration of B1 B cells.

The main function of B1 and MZ B cells is the T cell-independent production of antibodies against blood-borne particulate antigens (34, 35). Since RA signaling in B cells affects the frequency of MZ and B1 B cells, we analyzed whether the absence of RA signaling affects the T-independent humoral response. To address this, we immunized dnRARαCD19^Cre^ and control mice with TNP-Ficoll and collected serum at different time points to quantify anti-TNP antibodies by ELISA. We observed a decrease in both anti-TNP IgM and IgG3 titers in the dnRARαCD19^Cre^ mice compared to controls (Figures 3E,F). This reduction in Ag-specific antibody levels correlated with a reduction in anti-TNP IgM and anti-TNP IgG_3_ ASCs (Figures 3G,H). In contrast, no difference was observed in the number of anti-TNP-IgG_1_ ASC between dnRARαCD19^Cre^ and littermate control mice immunized with TNP-KLH and alum to induce a T-dependent humoral response (Figure 3I). In summary, these results suggest that RA signaling in B cells is important for the generation of T-independent humoral responses.

### RA Signaling in B Cells Does Not Affect B Cell Development in the Bone Marrow

**Figure 4:**
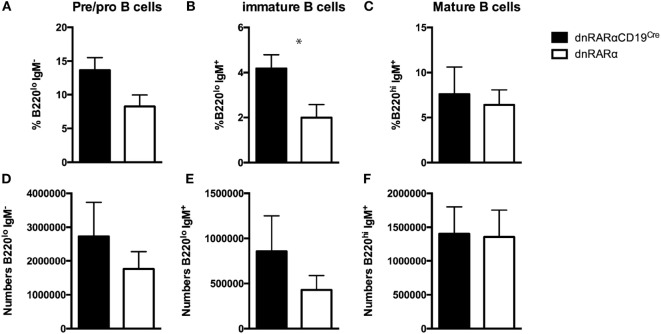
**RA signaling in B cells does not affect B cell development in the bone marrow**. Bone marrow from dnRARαCD19^Cre^ and control mice were obtained and pre/pro, immature and mature B cells were analyzed. **(A–C)** Percentage of the indicated B cell subsets of total live B cells in the bone marrow. **(D–F)** Absolute number. Three independent experiments with at least eight mice/group. *p < 0.05.

To assess whether RA signaling affects the development of B cells in the bone marrow, we quantified the percentage and absolute numbers of pre-B cells (B220^+^IgM^neg^IgD^neg^), immature B cells (B220^+^IgM^hi^IgD^neg^), and mature B cells (B220^+^IgM^int^IgD^+^) in dnRARαCD19^Cre^ mice, as previously described (29). Our results showed an increase in the percentage of immature B cells in dnRARαCD19^Cre^ mice compared with controls (Figure 4B). However, there was no difference in the absolute number of immature B cells (Figure 4E). In addition, neither the proportion nor the number of pre/pro B cells and mature B cells were affected by the abrogation of RA signaling in B cells (Figures 4A,C,D,F). These results indicate that B cell intrinsic RA signaling does not have an impact in the early development of B cells in the bone marrow.

## Discussion

Vitamin A deficiency has a major impact on T and B cell immunity. For example, deficiency in vitamin A drastically increases the mortality rate from measles (10) and the incidence of diarrhea (11). Moreover, supplementation in vitamin A or retinol reduces measles-related mortality and diarrhea in children (36, 37). Until now, the direct effect of RA on B cell function in protection against infection was unknown.

It has previously been published that human and mouse B cells express RARα (38); however, no detailed analysis of RAR expression in splenic B cells subsets has been reported. Our results show that RARα is expressed in all splenic B cell subsets; however, RARβ and γ isoforms are not expressed in these subsets. In addition, using a transgenic mouse model, in which RARα signaling is abrogated specifically in B cells, we demonstrate a profound defect in the development of MZ B cells and B1 B cells *in vivo*, which has a severe impact on T-independent humoral responses. This effect was not due to inhibition of the early development of B cells in the bone marrow, rather, RA signaling regulated the expression of S1P_1_ in T2 and MZ B cells and could, therefore, regulate MZ B cell development.

Our results demonstrated a reduction in the proportion of MZ B cells in the spleen and B1 B cells in the peritoneum when RA signaling was abrogated in B cells. Thus, we can hypothesize two different roles for RA; first, RA plays a role in B cell homing to the MZ and peritoneum, second, that RA regulates the development of both B1 and MZ B cells. It is well characterized that RA plays an important role in B cell migration to the gut by inducing the expression of α4β7 and CCR9 (24, 39). Thus, RA may also play a role in B cell migration to both MZ and peritoneum. It has previously been reported that integrins and S1P_1_ play an essential role in the localization of B cells to the MZ and peritoneum (28, 31, 40). Our results show that RA signaling is required for S1P_1_ expression in MZ B cells. In addition, we observed that chemotaxis to the ligand S1P is affected when RA signaling is abrogated in B cells. Therefore, our results support the idea that RA signaling promotes S1P_1_ expression in MZ B cells and, therefore, may affect the localization of B cells into the MZ.

Alternatively, RA may also play a role in B cell differentiation. It has been reported that RA inhibits B cell proliferation following BCR stimulation in both mouse and humans (41, 42). RA could play a role in reducing BCR signal strength in T2 B cells to promote MZ B cell differentiation, compared to FO B cells (43). This regulation could occur by three potential mechanisms; controlling the expression of negative regulators of BCR signaling (44), regulation of BAFF receptor, and NF-κB signaling (45) and/or be involved regulating Notch pathway (5).

Our results do not support a role for RA signaling in bone marrow development of B cells. MZ B cells are derived from bone marrow precursors (43); however, we found no changes in mature B cells in the bone marrow of dnRARαCD19^Cre^ mice, providing evidence that there is no effect of RA signaling on bone marrow B cell differentiation.

B1 B cells are developed prior to the first few weeks after birth (7). It has been demonstrated that B1 B cells can be reconstituted from fetal liver but not from bone marrow (46), suggesting that B1 B cell development takes place during embryogenesis. Our results showed a reduction in B1 B cells in the peritoneum of dnRARαCD19^Cre^ mice compared to controls, indicating a role for RA in B1 B cell development. NFATc1 has been implicated in this process (47) and NFATc1 expression in B1 B cells has been observed to be effected with vitamin A deficiency (21). Further studies to determine whether RAR binds directly to the NFATc1 promotor are needed.

Our results show that RA signaling in B cells is necessary for an effective T-independent B cell response, confirming previous studies showing vitamin A deficiency reduces T-independent responses to pneumococcal polysaccharides (48). Moreover, our results support several studies demonstrating the adjuvant impact of ATRA to enhance immunity.

Similarly to T cells (49), B cells can be re-educated in the acquisition of homing receptors in a RA-dependent manner (39). This plasticity could be used by naïve or memory B-cells to induce/inhibit migration to the spleen or other tissues. Such an effect has been demonstrated by He and collaborators, where intestinal epithelial cells and dendritic cells, which express retinal dehydrogenase enzymes and are able to produce RA, were shown to promote T-independent immune responses by regulating isotype switching from IgM or IgG to IgA plasma cells (50). Similarly, ATRA in combination with Vivotif results in increased IgA titers against typhoid (51).

Given the importance of RA in the migration and differentiation of B cells, it is relevant to determine which cells induce these properties on B lymphocytes. Dendritic cells from the lamina propria express a specific retinal dehydrogenase enzyme, which is essential for the synthesis of RA (52–54). Nonetheless, dendritic cells are not the only population of cells capable of synthesis of RA in the gut; macrophages and intestinal epithelial cells also express retinal dehydrogenase enzymes and are able to produce RA (53, 55, 56). However, it is unknown whether splenic macrophages play a role in the generation or migration of MZ B cells.

In conclusion, this study shows the important role of RA signaling in B cells for the generation of T-independent immune responses, with implications for the generation of new protocols for the use of ATRA as a novel adjuvant in vaccines.

## Author Contributions

RE and EM designed and performed research, collected and analyzed data, and wrote the manuscript; CO, EP and CB assisted in research. TW and GL provided critical reagents and assisted in research, RN and RE designed research and wrote the manuscript.

## Conflict of Interest Statement

The authors declare that the research was conducted in the absence of any commercial or financial relationships that could be construed as a potential conflict of interest.
